# Effects of Public Disclosure of a Japanese Celebrity's Oral Cancer: Trends in Oral Cancer Diagnoses

**DOI:** 10.1111/odi.15367

**Published:** 2025-05-15

**Authors:** Shihoko Koyama, Takahiro Tabuchi, Kayo Nakata, Toshitaka Morishima, Shuji Uchida, Miki Ishibashi, Isao Miyashiro

**Affiliations:** ^1^ Cancer Control Center Osaka International Cancer Institute Osaka Japan; ^2^ Department of Promoting Cooperation for Community Medicine, School of Medical Sciences Nagoya City University Nagoya Aichi Japan; ^3^ Division of Epidemiology, School of Public Health Tohoku University Graduate School of Medicine Sendai Japan; ^4^ Department of Oral and Maxillofacial Surgery, Graduate School of Dentistry Osaka University Osaka Japan; ^5^ Dental and Oral Surgery Izumi City General Hospital Osaka Japan; ^6^ Department of Cancer Oral Care, Dentistry and Oral maxillofacial Surgery Osaka International Cancer Institute Osaka Japan

**Keywords:** celebrity, epidemiology, Japan, oral cancer, the national population‐based cancer registry

## Abstract

**Objective:**

On February 19, 2019, Chiemi Hori, a renowned Japanese singer, publicly announced her diagnosis of stage IV tongue cancer. The news was widely reported by the Japanese media. This study examined the association between the celebrity's tongue cancer disclosure and trends in the number of oral cancer diagnoses in the national population‐based cancer registry (NCR) in Japan.

**Methods:**

Data from the NCR were used to plot monthly changes in the number of oral cancer diagnoses from 2016 to 2019 according to sex, age, residential area (divided into five categories based on the density of dentists), cancer site, and clinical stage at initial treatment. Time trends were analyzed using Joinpoint regression.

**Results:**

Between 2016 and 2019, 39,415 oral cancer cases were registered. After Ms. Hori's disclosure, oral cancer diagnoses increased significantly, while other head and neck cancers showed no significant changes. The number of oral cancer diagnoses increased from 784 in January 2019 to 1195 in March 2019 (an average monthly change rate of 8.6% from December 2018 to March 2019).

**Conclusions:**

There has been a sharp increase in the number of oral cancer diagnoses since the celebrity's disclosure. This suggests that it has raised public awareness of oral cancer.

**Abbreviations**
95% CI: 95% confidence interval
AMPC: average monthly percent change
MPC: monthly percent change

## 1 Introduction

On February 19, 2019, a famous Japanese singer, Chiemi Hori, announced on her blog that she had been diagnosed with stage IV oral cancer (squamous cell carcinoma of the left tongue) and that she had been admitted to hospital for surgery (Hori 2019). In the blog, she revealed that she had been visiting a dental clinic for the treatment of stomatitis since the previous summer but with no significant improvement; she finally visited a university hospital and was diagnosed with oral cancer (specifically tongue cancer). The news was widely reported by the Japanese media (The Asahi Shinbun 2019).

An increase in uptake of screening tests and information seeking has been observed after health‐related disclosures by celebrities. For example, when Angelina Jolie disclosed her decision to undergo a prophylactic bilateral mastectomy after learning that she had a deleterious BRCA1 mutation, referrals to cancer genetics clinics increased two‐ to threefold (Evans et al. 2014). In Japan, when the famous swimmer Ikee Rikako disclosed her leukemia diagnosis, an increase in Bone Marrow Donor Registration was seen (Yoshioka et al. 2021). A previous study showed that, among patients with oral cancer, some explicitly stated that their increased awareness of oral cancer from television news had led them to make an appointment with their general dentist (Grant et al. 2010). Google Trends data in Japan revealed a sharp increase in interest in the term “oral cancer” (Japanese: “koukuu‐gan”) between February 17 and 23, 2019 (Figure S1), suggesting a nationwide rise in searches.

While there is a growing body of evidence that celebrity cancer disclosures are associated with increased screening and testing, there is limited evidence to suggest an association with incidence. This was an ecological study which examined Chiemi Hori's oral cancer disclosure and trends in oral cancer diagnoses using data from the national population‐based cancer registry in Japan.

## 2 Materials and Methods

### 2.1 Data

We used data from the population‐based National Cancer Registry in Japan, which was established in 2016, enabling us to analyze nationwide trends in the number of oral cancer diagnoses (Ministry of Health). We have received data up to 2019 and utilized the data from 2016 to 2019. The Japan National Cancer Registry includes patient‐level information on sex, age (0–39, 40–49, 50–59, 60–69, 70–79, 80+), cancer site, clinical stage at first treatment, residential area, and month of diagnosis.

Residential area (patient's home prefecture) was categorized by ranking the 47 prefectures in Japan based on the number of dentists per 100,000 population. They were divided into five groups: highest (1st–10th), higher (11st–20th), middle (21st–30th), lower (31st–40th), and lowest (41st–47th). The highest group indicated the areas with the largest number of dentists.

The population‐based National Cancer Registry in Japan classifies tumors according to the third edition of the International Classification of Diseases for Oncology (ICD‐O‐3) (World Health Organization 2013). Regarding the analysis of the recorded diagnosis, the following oral and pharyngeal cancers were included ICD‐O‐3: 1.oral; 1‐1.tongue (ICD‐O‐3: C02.0, C02.1, C02.2, C02.3), 1‐2.gum (ICD‐O‐3: C03.0, C03.1, C03.9), 1‐3.floor of mouth (ICD‐O‐3: C04.0, C04.1, C04.8, C04.9, C05.0), 1‐4.inner lip and other and unspecified parts of mouth (other oral cancers) (ICD‐O‐3: C00.3, C00.4, C00.5, C00.6, C00.8, C00.9, C06.0, C06.1, C06.2, C06.8, C06.9), 2. External lip (ICD‐O‐3: C00.0, C00.1, C00.2), 3. Oropharynx (ICD‐O‐3: C01.9, C02.4, C05.1, C05.2, C09.0, C09.1, C09.8, C09.9, C10.0, C10.2, C10.3, C10.4, 10.8, C10.9), 4. Salivary (ICD‐O‐3: C07.9, C08.0, C08.1, C08.8, C08.9), 5. Nasopharynx (ICD‐O‐3: C11.0, C11.1, C11.2, C11.3, C11.8, C11.9), 6. Hypopharynx (ICD‐O‐3: C12.9, C13.0, C13.1, C13.2, C13.8, C13.9), 7. Other (ICD‐O‐3: C02.8, C02.9, C05.8, C05.9, C14.0, C14.2, C14.8) (Friz 2012; Thun et al. 2017). While the National Cancer Registry in Japan provides detailed histological data, our analysis concentrated on the variations in cancer diagnoses across different anatomical locations, and thus, histological typing was not performed.

### 2.2 Statistical Analyses

We conducted an ecological study. First, we summed the monthly counts of newly diagnosed oral cancer cases and compared pharyngeal cancers, which are typically aggregated with oral cancer and classified under the category of head and neck cancer. Second, we created figures and tables for the monthly number of oral cancer cases by detailed cancer site, sex, age, clinical stage at first treatment, and residential area. Time trends were analyzed using Joinpoint regression (version 5.0.2.0), which identifies time points where a statistically significant change in trend occurs (Kim et al. 2000). This approach was taken to avoid taking a predetermined cutoff point and to allow the Joinpoint regression to identify relevant time points. Joinpoint provides an estimate of the average monthly percentage change (AMPC), with 95% confidence intervals (95% CIs). Based on the observed high relative search volume on Google Search between February 17 and 23, 2019 (Figure S1), we conducted our research under the assumption that the fluctuations in search volume before and after this period were significantly influenced by the mass media.

## 3 Results

Between 2016 and 2019, we confirmed the registration of 91,406 cases of oral and pharyngeal cancer of which 39,415 were oral cancer cases.

**FIGURE 1:**
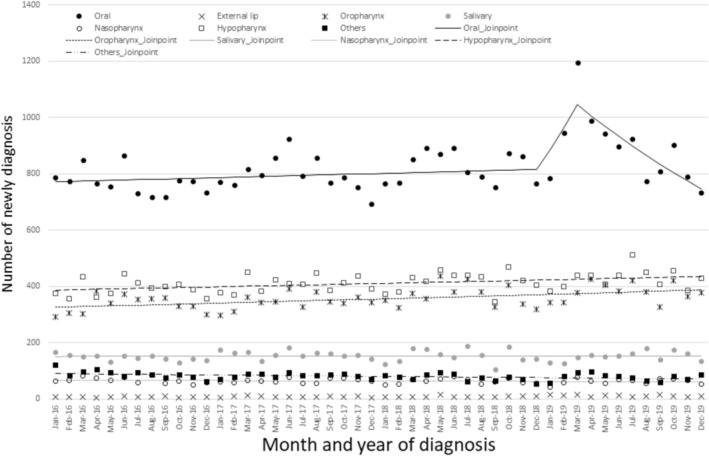
Monthly numbers of cases of newly diagnosed oral and pharyngeal cancer in 2016 and 2019 with Joinpoint regression.

**FIGURE 2:**
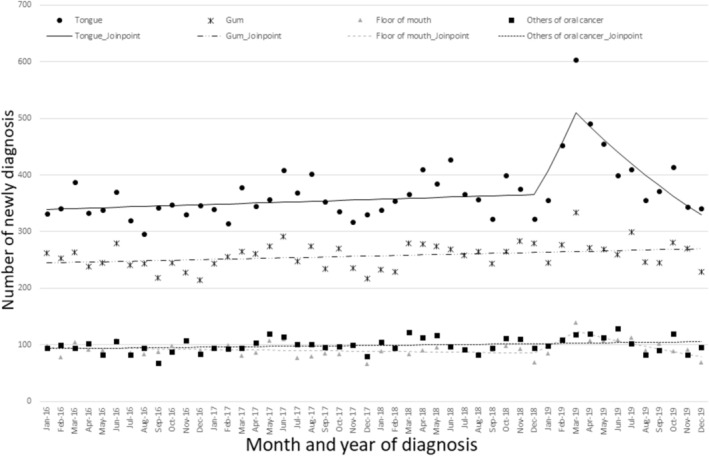
Monthly numbers of cases of newly diagnosed oral cancer in 2016 and 2019 with Joinpoint regression.

Figure 1 shows the monthly numbers of newly diagnosed oral and pharyngeal cancer cases in 2016 and 2019. The number of oral cancer diagnoses increased from 784 in January 2019 to 1195 in March 2019, a 1.5‐fold increase (Figure 1, Table S1). In the first quarter of 2019 (January–March 2019), before and after Ms. Hori announced her cancer (19 February 2019), oral cancer cases significantly increased (MPC: 8.60, 95% CI: 1.09– 11.61) (Figure 1, Table S2). In addition, when conducting a detailed analysis of oral cancer, the number of tongue and floor of mouth cancer cases among oral cancer cases significantly increased (tongue [MPC: 11.71, 95% CI: 2.56–15.39], floor of mouth [MPC: 13.32, 95% CI: 1.12–18.41]) (Figure 2, Tables S3 and S4).

**FIGURE 3:**
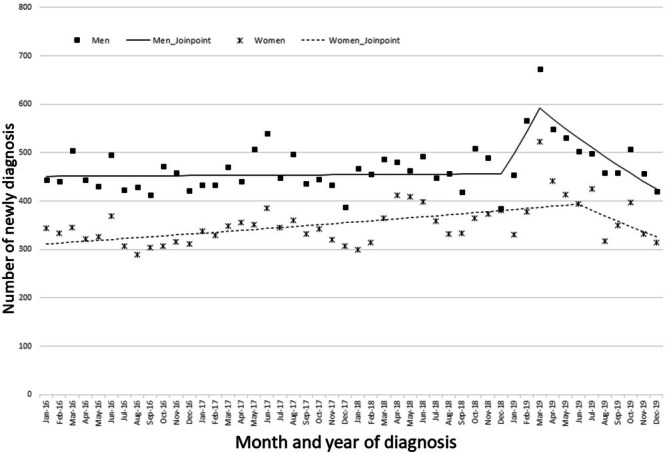
Trends of oral cancer diagnosis for each year by sex with Joinpoint regression.

**FIGURE 4:**
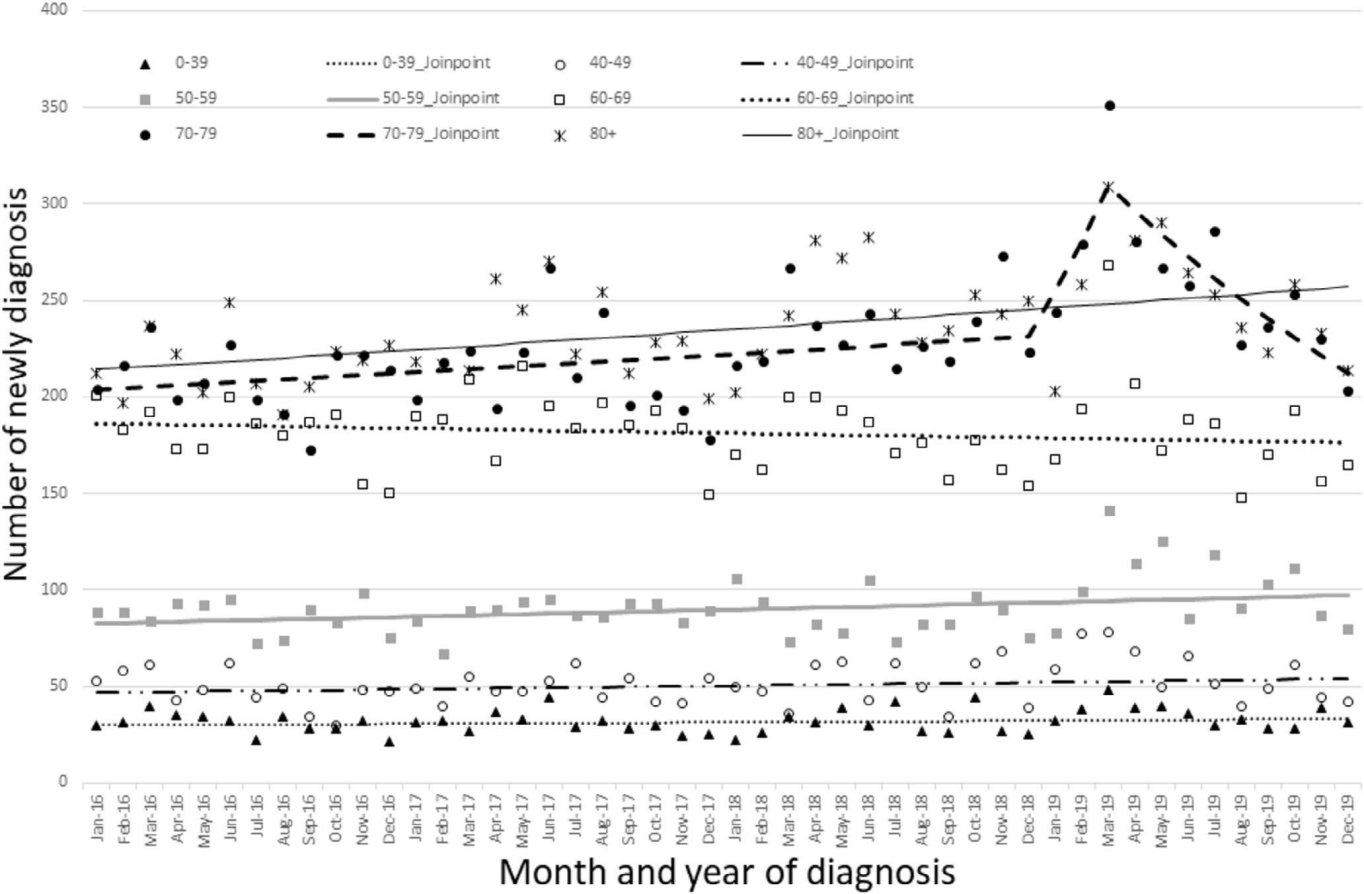
Trends of oral cancer diagnosis by age group with Joinpoint regression.

**FIGURE 5:**
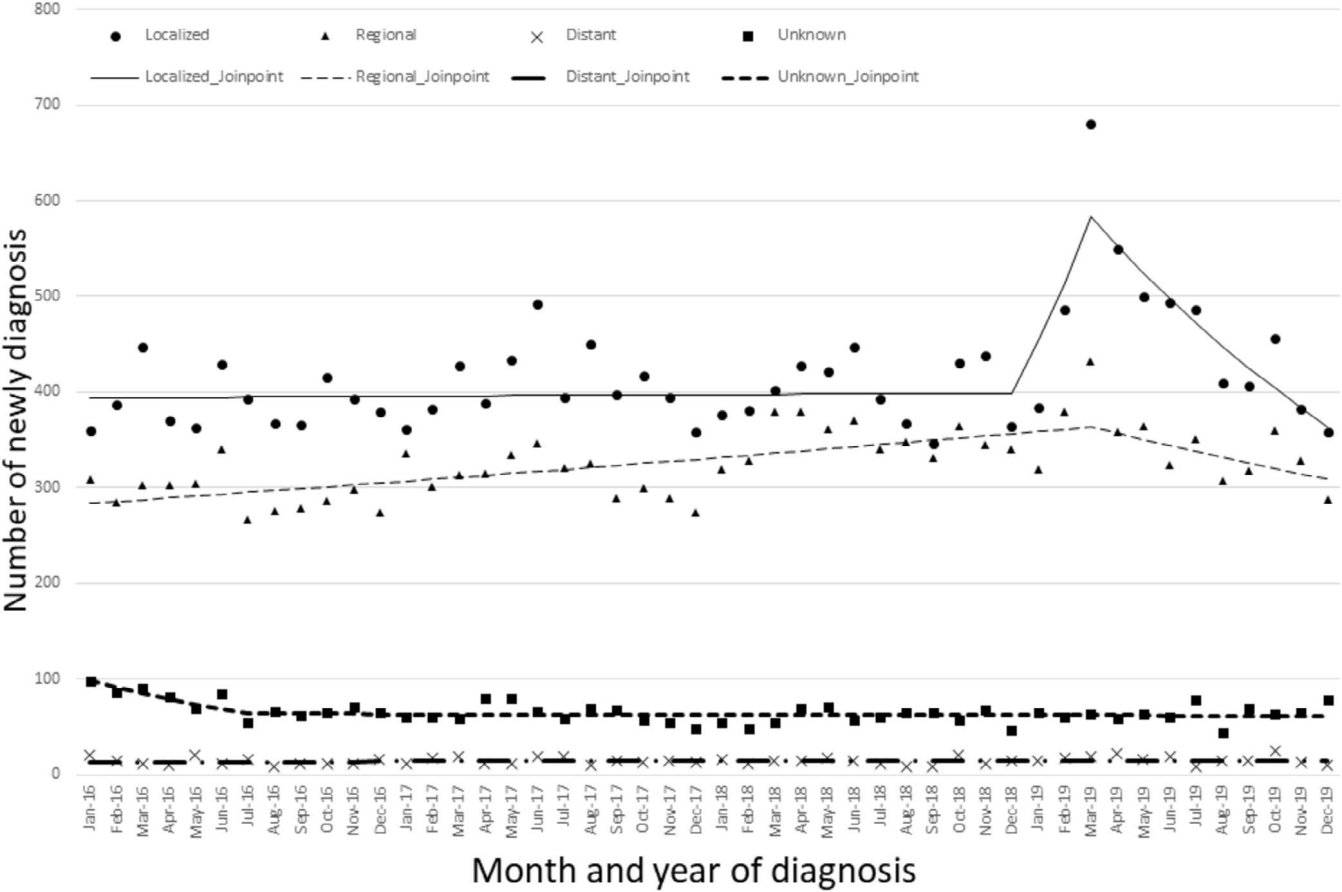
Change in oral cancer stage over a period of 4 years with Joinpoint regression.

We observed the trends in oral cancer for each month by sex (Figure 3). Among men, the number of oral cancer diagnoses increased significantly from December 2018 to March 2019 (MPC: 9.09, 95% CI: 1.14–12.31) (Table S3). An uptick in diagnoses was observed from December 2018 to March 2019, predominantly among individuals in their 70s and primarily for localized stages of cancer (70–79 age [MPC: 10.17, 95% CI, 1.12–14.09], localized stage [MPC: 13.52, 95% CI, 3.08–17.46]) (Figures 4 and 5, Table S3). Regarding cancer stage, there was no surge in distant or regional metastasis around February 2019. However, there was a sharp increase in oral cancer diagnoses between December 2018 and March 2019, but only for localized cancer (Figure 5, Table S3). The number of diagnoses in March 2019 reached its peak across all age categories (Table S4).

**FIGURE 6:**
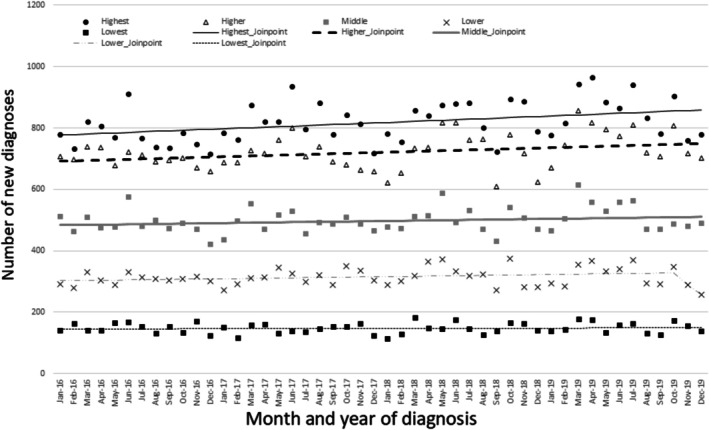
Temporal trends in oral cancer diagnosis based on dentist population density by prefecture of patient residence with Joinpoint regression.

In terms of residential area, there was no change in the number of cases in any group before and after Ms. Hori announced her oral cancer (19 February 2019) (Figure 6, Table S3).

## 4 Discussion

Our findings revealed that after Ms. Hori announced her oral cancer diagnosis on 19 February 2019, there was a steep increase in oral cancer diagnoses, although pharyngeal cancers showed no significant changes. Similar trends were observed in tongue and floor of mouth cancers among oral cancers. Notably, the number of men in their 70s with localized cancer increased rapidly during this period.

Previous studies have shown that media coverage of cancer‐related events, such as the announcement of a celebrity's cancer or a cancer‐related death, leads to an increase of interest in cancer screening and internet searches for the cancer in question (Evans et al. 2014; Juthe et al. 2015; Kaleem et al. 2019; Rahmani et al. 2018; Yoshioka et al. 2021). From our research, it has become clear that the number of diagnoses increases along with the growing interest in cancer. After Ms. Hori's public announcement of her oral cancer, there has been a significant surge in the number of oral cancer diagnoses. In particular, regarding the localized stage at diagnosis, the MPC significantly increased to 13.52 between December 2018 and March 2019. A survey in the United States revealed a lack of public knowledge regarding head and neck cancer, with limited recognition of symptoms. These findings suggest that improving public awareness is crucial for early detection and better treatment outcomes (Luryi et al. 2014). It is evident that public disclosure by prominent figures and their subsequent treatment for head and neck cancer have contributed to the advancement of treatment, preventive measures, and public knowledge regarding these cancers (Folz et al. 2007; Martinez‐Ramirez et al. 2024). This remarkably high MPC strongly indicates a surge in diagnoses of localized stage oral cancer, likely driven by media coverage.

It is possible that the increased interest in oral cancer, as observed in the studies cited above, led to the significant increase in the number of diagnoses identified in our study via two pathways. The first is that the increased social interest in oral cancer may have led to the general public observing their oral health more closely. Although oral cancer screening is not widely available in Japan, it is possible that increased awareness encouraged people to visit dental clinics or other medical institutions. Previous research has shown that media coverage of prominent figures can lead to an increase in secondary prevention (Evans et al. 2014). In addition, Takeda et al.'s (2022) study found, from a monthly dental insurance claims report from 2017 to 2020, that March 2019 had the highest number of claims (Takeda et al. 2022). The second possible pathway is that diagnosing general physicians and general dentists may have conducted more detailed examinations than usual. In Ms. Hori's blog, she reported that she had experienced a delay in diagnosis by healthcare professionals, despite visiting the dentist for a year, and that she was not diagnosed with oral cancer until it reached stage IV. Oral cancer is known to be subject to “professional delay,” which refers to delayed referrals from general physicians and dentists to specialized medical and dental practitioners, leading to delays in diagnosis (Lima et al. 2021; Onizawa et al. 2003). As oral cancer is rarely found in general dental practice, dentists may not immediately suspect malignancy. The accounts of Ms. Hori's cancer likely led to a more comprehensive approach to diagnosing oral cancer, which may have resulted in its early detection. From these findings, it can be surmised that many more cases are diagnosed at the localized stage, and the number of locations that are difficult to detect during normal dental treatment but are now recognized, such as floor of mouse cancer, has increased rapidly.

Smoking and alcohol consumption are recognized as major risk factors for the development of oral cancer. In Japan, annual data on smoking and alcohol consumption were available (Cancer information service in Japan). The smoking rate among Japanese men showed a decreasing trend between 1995 and 2019. Regarding alcohol consumption, the prevalence of habitual drinkers (those who consume alcohol three or more times per week) among men significantly decreased from 51.5% in 1989 to 33.9% in 2019 (Ministry of Health). These trends align with the overall decrease observed in men, indicated by an AMPC of −0.13 (Table S3). Conversely, the smoking rate among women remained relatively stable between 2004 and 2019. The prevalence of habitual drinkers among women increased from 6.3% in 1989 to 8.8% in 2019. This increase is consistent with the significant AMPC of 0.09 observed in women between 2016 and 2019 (Table S3).

The results of this study showed that there was little change in the number of diagnoses of oral cancer by region, regardless of the density of dentists. The presence or absence of a dental school was a major factor for differences in the number of dentists relative to the population in Japan (Hashimura et al. 2019). However, it is likely that media coverage of oral cancer in celebrities provided information in every region, regardless of these differences. Oral cancer is one of the rarest types of cancer, and many people were unaware of its existence prior to Ms. Hori's diagnosis.

The present study has some limitations. First, because we used the monthly data for oral cancer diagnosis, it cannot be conclusively stated that the increase in diagnosis is solely due to the publicity given to a prominent figure such as Ms. Hori. We also believe that as the current cancer registry data only extend to 2019, future research should include data from beyond that date in order to observe and consider long‐term trends in oral cancer diagnosis. Second, the possibility of overdiagnosis should be considered (Parak et al. 2022). Overdiagnosis occurs when a condition that would not have progressed or affected the patient's health is detected through testing or screening, raising the risk of unnecessary treatment, side effects, and potential psychological stress. From our study findings, particularly concerning the increased diagnoses of localized cancers, we consider it necessary to examine mortality data in the future to determine whether these cases were genuinely early detections or instances of overdiagnosis.

Our study showed that a celebrity disclosure was followed by a sharp increase in oral cancer diagnoses. The disclosure may have increased awareness of cancers in the general population, and healthcare professionals may have become overzealous in making diagnoses.

## Author Contributions

**Shihoko Koyama:** writing – review and editing, writing – original draft, visualization, conceptualization, data curation, formal analysis, funding acquisition, investigation, methodology, project administration, resources, software, validation. **Takahiro Tabuchi:** writing – review and editing, conceptualization, methodology, supervision, validation. **Kayo Nakata:** writing – review and editing, conceptualization, supervision, validation. **Toshitaka Morishima:** validation, writing – review and editing. **Shuji Uchida:** writing – review and editing. **Miki Ishibashi:** writing – review and editing. **Isao Miyashiro:** writing – review and editing.

## Ethics Statement

This study comprised investigative research in accordance with the Cancer Registry Act. We received the registry information in accordance with the law and independently created and processed the provided aggregate/statistical information. The protocol was reviewed by the Data Utilization Committee of the National Cancer Registration Office according to the procedure stipulated by law (A2022‐0031). According to the rules of the Japan National Cancer Registry, if there are fewer than nine cases, the number cannot be explicitly stated for reasons of patient confidentiality.

Approval of the research protocol by an Institutional Reviewer Board: the Research Ethics Committee of the Osaka International Cancer Institute (No. 21123‐2). It is mandatory to obtain ethical review board approval at each participating institution for the use of National Cancer Registry data.

## Consent

The authors have nothing to report.

## Conflicts of Interest

The authors declare no conflicts of interest.

## Supporting information


**Figure S1.** Google trends in Japan; search terms for “oral cancer” in Japanese from January 2016 (27/12/2015) to December 2019 (27/12/2019).
**Table S1.** Monthly breakdown of oral and pharyngeal cancer cases by site (*n* = 91,406).
**Table S2.** Joinpoint trend analyses in oral and pharyngeal cancer cases (*n* = 91,406).
**Table S3.** Joinpoint trend analyses in oral cancer cases (*n* = 39,415).
**Table S4.** Monthly number of oral cancer diagnosis by category (*n* = 39,415).

## Data Availability

The data that support the findings of this study are available on request from the corresponding author. The data are not publicly available due to privacy or ethical restrictions.
