# Supplementary material for: Effects of Public Disclosure of a Japanese Celebrity's Oral Cancer: Trends in Oral Cancer Diagnoses
Source: Oral Dis. 2025 May 15;31(10):2878–84. doi: 10.1111/odi.15367 (PMC12721700; doi:10.1111/odi.15367)
Supplement: Supplementary file 1 — Figure S1. Google trends in Japan; search terms for “oral cancer” in Japanese from January 2016 (27/12/2015) to December 2019 (27/12/2019). Table S1. Monthly breakdown of oral and pharyngeal cancer cases by site (n = 91,406). Table S2. Joinpoint trend analyses in oral and pharyngeal cancer cases (n = 91,406). Table S3. Joinpoint trend analyses in oral cancer cases (n = 39,415). Table S4. Monthly number of oral cancer diagnosis by category (n = 39,415). [file ODI-31-2878-s001.docx]

**Supporting Information**:

Figure S1. Google trends in Japan; searches for “oral cancer” in Japanese from Jan 2016 (27/12/2015) to Dec 2019 (27/12/2019)

Table S1. Monthly breakdown of oral and pharyngeal cancer cases by site (n=91,406)

|  | Oral and pharyngeal cancer site^a^ | | | | | | | | | |
| --- | --- | --- | --- | --- | --- | --- | --- | --- | --- | --- |
|  | Oral | Oral | | | | Oropharynx | Salivary | Nasopharynx | Hypopharynx | Other |
|  |  | Tongue | Gum | Floor of mouth | Other oral cancers |  |  |  |  |  |
| Jan-16 | 788 | 332 | 262 | 99 | 95 | 293 | 168 | 63 | 376 | 123 |
| Feb-16 | 773 | 341 | 253 | 79 | 100 | 307 | 158 | 68 | 357 | 84 |
| Mar-16 | 850 | 387 | 263 | 105 | 95 | 303 | 151 | 82 | 434 | 97 |
| Apr-16 | 765 | 333 | 238 | 92 | 102 | 384 | 153 | 74 | 361 | 106 |
| May-16 | 756 | 339 | 245 | 90 | 82 | 341 | 132 | 68 | 376 | 96 |
| Jun-16 | 865 | 370 | 280 | 109 | 106 | 373 | 155 | 78 | 445 | 82 |
| Jul-16 | 730 | 320 | 241 | 87 | 82 | 353 | 145 | 58 | 413 | 94 |
| Aug-16 | 719 | 296 | 244 | 84 | 95 | 358 | 154 | 86 | 395 | 88 |
| Sep-16 | 717 | 343 | 218 | 88 | 68 | 360 | 143 | 57 | 400 | 75 |
| Oct-16 | 778 | 347 | 245 | 98 | 88 | 329 | 130 | 65 | 408 | 88 |
| Nov-16 | 774 | 331 | 227 | 108 | 108 | 329 | 143 | 52 | 389 | 78 |
| Dec-16 | 734 | 346 | 214 | 90 | 84 | 300 | 139 | 60 | 358 | 64 |
| Jan-17 | 771 | 340 | 244 | 93 | 94 | 297 | 175 | 61 | 379 | 70 |
| Feb-17 | 762 | 314 | 255 | 100 | 93 | 311 | 164 | 58 | 370 | 79 |
| Mar-17 | 818 | 378 | 265 | 81 | 94 | 363 | 167 | 66 | 450 | 89 |
| Apr-17 | 796 | 345 | 261 | 87 | 103 | 343 | 135 | 64 | 384 | 90 |
| May-17 | 858 | 357 | 274 | 108 | 119 | 346 | 157 | 62 | 424 | 78 |
| Jun-17 | 924 | 409 | 291 | 110 | 114 | 391 | 184 | 78 | 411 | 95 |
| Jul-17 | 794 | 369 | 247 | 77 | 101 | 326 | 153 | 57 | 409 | 84 |
| Aug-17 | 857 | 402 | 274 | 80 | 101 | 382 | 166 | 56 | 448 | 85 |
| Sep-17 | 768 | 353 | 234 | 85 | 96 | 346 | 163 | 75 | 386 | 88 |
| Oct-17 | 787 | 336 | 270 | 84 | 97 | 341 | 154 | 75 | 414 | 89 |
| Nov-17 | 754 | 317 | 236 | 101 | 100 | 361 | 157 | 70 | 438 | 82 |
| Dec-17 | 694 | 331 | 217 | 66 | 80 | 343 | 144 | 65 | 391 | 71 |
| Jan-18 | 766 | 339 | 233 | 89 | 105 | 351 | 125 | 50 | 372 | 84 |
| Feb-18 | 770 | 354 | 229 | 93 | 94 | 325 | 136 | 54 | 380 | 80 |
| Mar-18 | 852 | 366 | 280 | 84 | 122 | 375 | 180 | 69 | 432 | 72 |
| Apr-18 | 892 | 410 | 278 | 91 | 113 | 356 | 179 | 64 | 419 | 88 |
| May-18 | 872 | 385 | 274 | 96 | 117 | 436 | 159 | 73 | 459 | 95 |
| Jun-18 | 891 | 427 | 269 | 98 | 97 | 381 | 149 | 81 | 441 | 90 |
| Jul-18 | 806 | 366 | 258 | 90 | 92 | 427 | 189 | 69 | 440 | 63 |
| Aug-18 | 789 | 357 | 265 | 84 | 83 | 382 | 156 | 53 | 435 | 76 |
| Sep-18 | 752 | 323 | 243 | 92 | 94 | 326 | 107 | 61 | 346 | 65 |
| Oct-18 | 873 | 399 | 265 | 98 | 111 | 406 | 186 | 75 | 469 | 79 |
| Nov-18 | 863 | 376 | 284 | 93 | 110 | 338 | 142 | 58 | 422 | 72 |
| Dec-18 | 766 | 322 | 280 | 69 | 95 | 320 | 143 | 56 | 406 | 56 |
| Jan-19 | 784 | 355 | 245 | 85 | 99 | 343 | 131 | 43 | 384 | 58 |
| Feb-19 | 945 | 452 | 277 | 107 | 109 | 343 | 126 | 58 | 401 | 82 |
| Mar-19 | 1,195 | 603 | 334 | 140 | 118 | 378 | 150 | 77 | 439 | 94 |
| Apr-19 | 990 | 491 | 272 | 107 | 120 | 426 | 156 | 63 | 441 | 99 |
| May-19 | 944 | 455 | 269 | 107 | 113 | 406 | 151 | 55 | 409 | 84 |
| Jun-19 | 897 | 399 | 260 | 109 | 129 | 383 | 154 | 69 | 440 | 82 |
| Jul-19 | 924 | 410 | 299 | 113 | 102 | 420 | 162 | 68 | 512 | 76 |
| Aug-19 | 775 | 356 | 246 | 90 | 83 | 380 | 181 | 56 | 451 | 65 |
| Sep-19 | 809 | 372 | 245 | 102 | 90 | 328 | 141 | 73 | 407 | 59 |
| Oct-19 | 904 | 414 | 281 | 89 | 120 | 421 | 176 | 71 | 457 | 82 |
| Nov-19 | 789 | 344 | 270 | 92 | 83 | 364 | 161 | 66 | 385 | 70 |
| Dec-19 | 735 | 341 | 229 | 69 | 96 | 379 | 134 | 54 | 428 | 86 |

^a^ Removed external lip due to low number of cases

Table S2. Joinpoint trend analyses in oral and pharyngeal cancer cases (n=91,406)

|  | (A)MPC [95%CI] | | | | | |
| --- | --- | --- | --- | --- | --- | --- |
| Oral (full range) | -0.074 | [ | -0.364 | ; | 0.1222 | ] |
| 1: Jan 16-Den 18 | 0.1575 | [ | -0.2748 | ; | 0.3874 | ] |
| 2: Dec 18-Mar 19 | 8.6003 | [ | 1.086 | ; | 11.6131 | ] |
| 3: Mar 19-Dec 19 | -3.6797 | [ | -6.6892 | ; | -2.2315 | ] |
| Oropharynx | 0.3666 | [ | 0.1454 | ; | 0.5923 | ] |
| Salivary | 0.0091 | [ | -0.253 | ; | 0.2745 | ] |
| Nasopharynx | -0.1879 | [ | -0.5506 | ; | 0.1843 | ] |
| Hypopharynx | 0.2499 | [ | 0.078 | ; | 0.423 | ] |
| Other | -0.4927 | [ | -0.8102 | ; | -0.1773 | ] |

AMPC (for full trends): Average Monthly Percent Change

MPC: Monthly Percent Change

Table S3. Joinpoint trend analyses in oral cancer cases (n=39,415)

|  | (A)MPC [95%CI] | | | | | |
| --- | --- | --- | --- | --- | --- | --- |
| **Oral cancer** |  |  |  |  |  |  |
| Tongue (full range) | -0.06 | [ | -0.3552 | ; | 0.1685 | ] |
| 1: Jan 16-Den 18 | 0.2125 | [ | -0.1598 | ; | 0.4656 | ] |
| 2: Dec 18-Mar 19 | 11.7082 | [ | 2.5563 | ; | 15.3904 | ] |
| 3: Mar 19-Dec 19 | -4.7148 | [ | -7.7418 | ; | -3.0304 | ] |
| Gum | 0.2073 | [ | 0.0195 | ; | 0.3966 | ] |
| Floor of mouth (full range) | -0.3754^a^ | [ | -0.8769 | ; | -0.0633 | ] |
| 1: Jan 16-Den 18 | -0.2966 | [ | -1.1192 | ; | 0.1079 | ] |
| 2: Dec 18-Mar 19 | 13.3189 | [ | 1.1154 | ; | 18.4089 | ] |
| 3: Mar 19-Dec 19 | -4.8546 | [ | -10.3097 | ; | -2.4953 | ] |
| Other oral cancers | 0.2639 | [ | -0.0228 | ; | 0.5507 | ] |
| **Sex** |  |  |  |  |  |  |
| Men (full range) | -0.1279 | [ | -0.4417 | ; | 0.0802 | ] |
| 1: Jan 16-Den 18 | 0.0322 | [ | -0.4348 | ; | 0.2791 | ] |
| 2: Dec 18-Mar 19 | 9.0890^a^ | [ | 1.1417 | ; | 12.3075 | ] |
| 3: Mar 19-Dec 19 | -3.6260^a^ | [ | -7.0066 | ; | -2.0817 | ] |
| Women (full range) | 0.0944 | [ | -0.3352 | ; | 0.6601 | ] |
| 1: Jan 16-Jun 19 | 0.5702^a^ | [ | 0.2518 | ; | 3.7238 | ] |
| 2: Jun 19-Dec 19 | -3.0967 | [ | -14.3309 | ; | 0.3947 | ] |
| **Age** |  |  |  |  |  |  |
| 0-39 | 0.2296 | [ | -0.2165 | ; | 0.6835 | ] |
| 40-49 | 0.3012 | [ | -0.2077 | ; | 0.8208 | ] |
| 50-59 | 0.3461^a^ | [ | 0.0301 | ; | 0.6701 | ] |
| 60-69 | -0.1158 | [ | -0.3599 | ; | 0.1323 | ] |
| 70-79 (full range) | 0.0841 | [ | -0.2919 | ; | 0.3368 | ] |
| 1: Jan 16-Den 18 | 0.3636 | [ | -0.3588 | ; | 0.6635 | ] |
| 2: Dec 18-Mar 19 | 10.1697^a^ | [ | 1.1169 | ; | 14.086 | ] |
| 3: Mar 19-Dec 19 | -4.1142^a^ | [ | -8.2657 | ; | -2.2413 | ] |
| 80+ | 0.3883^a^ | [ | 0.1633 | ; | 0.6151 | ] |
| **Stage** |  |  |  |  |  |  |
| Localized (full range) | -0.1708 | [ | -0.5244 | ; | 0.0677 | ] |
| 1: Jan 16-Den 18 | 0.0358 | [ | -0.3808 | ; | 0.3236 | ] |
| 2: Dec 18-Mar 19 | 13.5183^a^ | [ | 3.0819 | ; | 17.4594 | ] |
| 3: Mar 19-Dec 19 | -5.1225^a^ | [ | -8.3359 | ; | -3.3635 | ] |
| Regional (full range) | 0.1791 | [ | -0.1981 | ; | 0.5425 | ] |
| 1: Jan 16-Mar 19 | 0.6535^a^ | [ | 0.4217 | ; | 1.5062 | ] |
| 2: Mar 19-Dec 19 | -1.7994 | [ | -9.2452 | ; | 0.1255 | ] |
| Distant | 0.2277 | [ | -0.4208 | ; | 0.8942 | ] |
| Unknown (full range) | -1.0019^a^ | [ | -1.5032 | ; | -0.4053 | ] |
| 1: Jan 16- Jul 16 | -7.0687^a^ | [ | -21.3772 | ; | -1.3104 | ] |
| 2: Jul 16-Dec 19 | -0.0814 | [ | -0.4466 | ; | 0.6152 | ] |
| **Density of dentists per population** | | | |  |  |  |
| Highest | 0.2127^a^ | [ | 0.034 | ; | 0.3956 | ] |
| Higher | 0.1697^a^ | [ | 0.0029 | ; | 0.3377 | ] |
| Middle | 0.116 | [ | -0.0606 | ; | 0.2925 | ] |
| Lower (full range) | -0.3534 | [ | -0.7618 | ; | 0.3036 | ] |
| 1: Jan 16- Oct 19 | 0.1792 | [ | -0.0953 | ; | 2.5025 | ] |
| 2: Oct 19-Dec 19 | -11.6145 | [ | -19.5806 | ; | 0.1189 | ] |
| Lowest | 0.0551 | [ | -0.2295 | ; | 0.3433 | ] |

AMPC (for full trends): Average Monthly Percent Change

MPC: Monthly Percent Change

^a^ p<0.05

Table S4. Monthly number of oral cancer diagnoses by category (n=39,415)

|  | Sex | | Age | | | | | | Stage | | | Density of dentists per population | | | | |
| --- | --- | --- | --- | --- | --- | --- | --- | --- | --- | --- | --- | --- | --- | --- | --- | --- |
|  | Men | Women | 0-39 | 40-49 | 50-59 | 60-69 | 70-79 | 80+ | Localized | Regional | Distant | Highest | Higher | Middle | Lower | Lowest |
|  |  |  |  |  |  |  |  |  |  |  |  |  |  |  |  |  |
| Jan-16 | 444 | 344 | 30 | 53 | 88 | 201 | 204 | 212 | 360 | 309 | 21 | 779 | 707 | 511 | 291 | 142 |
| Feb-16 | 440 | 333 | 31 | 58 | 88 | 183 | 216 | 197 | 387 | 285 | 15 | 732 | 698 | 462 | 280 | 162 |
| Mar-16 | 504 | 346 | 40 | 61 | 84 | 192 | 236 | 237 | 447 | 302 | 11 | 820 | 740 | 509 | 331 | 142 |
| Apr-16 | 443 | 322 | 35 | 43 | 93 | 173 | 199 | 222 | 371 | 303 | 10 | 807 | 737 | 475 | 304 | 142 |
| May-16 | 430 | 326 | 34 | 48 | 92 | 173 | 207 | 202 | 363 | 304 | 20 | 770 | 677 | 476 | 290 | 165 |
| Jun-16 | 496 | 369 | 32 | 62 | 95 | 200 | 227 | 249 | 429 | 341 | 11 | 911 | 721 | 575 | 331 | 167 |
| Jul-16 | 423 | 307 | 22 | 44 | 72 | 186 | 199 | 207 | 393 | 266 | 16 | 768 | 712 | 479 | 314 | 153 |
| Aug-16 | 429 | 290 | 34 | 49 | 74 | 180 | 191 | 191 | 367 | 276 | - | 737 | 689 | 499 | 309 | 132 |
| Sep-16 | 413 | 304 | 28 | 34 | 90 | 187 | 173 | 205 | 366 | 278 | 11 | 736 | 696 | 473 | 303 | 153 |
| Oct-16 | 471 | 307 | 28 | 30 | 83 | 191 | 222 | 224 | 416 | 286 | 11 | 785 | 702 | 489 | 308 | 133 |
| Nov-16 | 458 | 316 | 32 | 48 | 98 | 155 | 222 | 219 | 393 | 298 | 12 | 748 | 670 | 469 | 316 | 170 |
| Dec-16 | 422 | 312 | 21 | 47 | 75 | 150 | 214 | 227 | 379 | 274 | 16 | 715 | 659 | 421 | 301 | 124 |
| Jan-17 | 433 | 338 | 31 | 49 | 84 | 190 | 199 | 218 | 362 | 336 | 12 | 784 | 687 | 435 | 273 | 151 |
| Feb-17 | 433 | 329 | 32 | 40 | 67 | 188 | 218 | 217 | 383 | 301 | 18 | 762 | 688 | 496 | 292 | 116 |
| Mar-17 | 470 | 348 | 27 | 55 | 89 | 209 | 224 | 214 | 427 | 313 | 19 | 874 | 726 | 552 | 312 | 158 |
| Apr-17 | 440 | 356 | 37 | 47 | 90 | 167 | 194 | 261 | 389 | 315 | 12 | 821 | 717 | 470 | 313 | 161 |
| May-17 | 507 | 351 | 33 | 47 | 94 | 216 | 223 | 245 | 433 | 334 | 11 | 820 | 760 | 517 | 345 | 130 |
| Jun-17 | 539 | 385 | 44 | 53 | 95 | 195 | 267 | 270 | 492 | 346 | 19 | 936 | 801 | 528 | 326 | 138 |
| Jul-17 | 448 | 346 | 29 | 62 | 87 | 184 | 210 | 222 | 395 | 321 | 19 | 796 | 708 | 454 | 299 | 136 |
| Aug-17 | 497 | 360 | 32 | 44 | 86 | 197 | 244 | 254 | 451 | 326 | 10 | 881 | 739 | 493 | 320 | 147 |
| Sep-17 | 436 | 332 | 28 | 54 | 93 | 185 | 196 | 212 | 397 | 289 | 14 | 779 | 690 | 486 | 290 | 152 |
| Oct-17 | 445 | 342 | 30 | 42 | 93 | 193 | 201 | 228 | 417 | 300 | 13 | 844 | 680 | 509 | 350 | 153 |
| Nov-17 | 434 | 320 | 24 | 41 | 83 | 184 | 193 | 229 | 395 | 289 | 15 | 813 | 663 | 488 | 336 | 164 |
| Dec-17 | 387 | 307 | 25 | 54 | 89 | 149 | 178 | 199 | 359 | 274 | 13 | 717 | 658 | 464 | 304 | 123 |
| Jan-18 | 467 | 299 | 22 | 50 | 106 | 170 | 216 | 202 | 376 | 319 | 16 | 781 | 622 | 478 | 290 | 114 |
| Feb-18 | 455 | 315 | 26 | 47 | 94 | 162 | 219 | 222 | 381 | 329 | 12 | 756 | 653 | 472 | 300 | 129 |
| Mar-18 | 487 | 365 | 34 | 36 | 73 | 200 | 267 | 242 | 402 | 380 | 15 | 858 | 734 | 512 | 318 | 182 |
| Apr-18 | 480 | 412 | 31 | 61 | 82 | 200 | 237 | 281 | 428 | 379 | 15 | 840 | 737 | 515 | 365 | 149 |
| May-18 | 463 | 409 | 39 | 63 | 78 | 193 | 227 | 272 | 422 | 361 | 18 | 875 | 816 | 587 | 372 | 146 |
| Jun-18 | 492 | 399 | 30 | 43 | 105 | 187 | 243 | 283 | 447 | 371 | 15 | 879 | 818 | 492 | 333 | 175 |
| Jul-18 | 448 | 358 | 42 | 62 | 73 | 171 | 215 | 243 | 393 | 341 | 12 | 882 | 762 | 531 | 318 | 146 |
| Aug-18 | 457 | 332 | 27 | 50 | 82 | 176 | 226 | 228 | 367 | 348 | - | 801 | 763 | 471 | 324 | 127 |
| Sep-18 | 419 | 333 | 26 | 34 | 82 | 157 | 219 | 234 | 347 | 331 | - | 724 | 610 | 431 | 273 | 138 |
| Oct-18 | 508 | 365 | 44 | 62 | 97 | 178 | 239 | 253 | 430 | 365 | 20 | 893 | 779 | 542 | 375 | 166 |
| Nov-18 | 490 | 373 | 27 | 68 | 90 | 162 | 273 | 243 | 439 | 345 | 11 | 887 | 717 | 506 | 281 | 162 |
| Dec-18 | 385 | 381 | 25 | 39 | 75 | 154 | 223 | 250 | 364 | 340 | 15 | 788 | 625 | 469 | 281 | 141 |
| Jan-19 | 454 | 330 | 32 | 59 | 78 | 168 | 244 | 203 | 384 | 320 | 15 | 776 | 670 | 465 | 293 | 138 |
| Feb-19 | 567 | 378 | 38 | 77 | 99 | 194 | 279 | 258 | 487 | 380 | 17 | 817 | 745 | 503 | 285 | 144 |
| Mar-19 | 673 | 522 | 48 | 78 | 141 | 268 | 351 | 309 | 680 | 432 | 19 | 944 | 856 | 615 | 355 | 178 |
| Apr-19 | 548 | 442 | 39 | 68 | 114 | 207 | 281 | 281 | 550 | 359 | 22 | 965 | 818 | 559 | 368 | 176 |
| May-19 | 531 | 413 | 40 | 50 | 125 | 172 | 267 | 290 | 500 | 365 | 16 | 884 | 795 | 528 | 333 | 134 |
| Jun-19 | 503 | 394 | 36 | 66 | 85 | 188 | 258 | 264 | 494 | 324 | 19 | 865 | 772 | 558 | 340 | 158 |
| Jul-19 | 499 | 425 | 30 | 51 | 118 | 186 | 286 | 253 | 486 | 351 | - | 941 | 810 | 563 | 370 | 162 |
| Aug-19 | 458 | 317 | 33 | 40 | 91 | 148 | 227 | 236 | 410 | 307 | 14 | 834 | 720 | 471 | 294 | 132 |
| Sep-19 | 459 | 350 | 28 | 49 | 103 | 170 | 236 | 223 | 407 | 318 | 14 | 781 | 707 | 470 | 292 | 127 |
| Oct-19 | 507 | 397 | 28 | 61 | 111 | 193 | 253 | 258 | 456 | 360 | 25 | 904 | 808 | 486 | 348 | 173 |
| Nov-19 | 457 | 332 | 39 | 44 | 87 | 156 | 230 | 233 | 383 | 328 | 13 | 759 | 717 | 480 | 288 | 155 |
| Dec-19 | 420 | 315 | 31 | 42 | 80 | 165 | 203 | 214 | 359 | 287 | 10 | 780 | 703 | 490 | 257 | 138 |

"-" According to the rules of the Japan National Cancer Registry, the published value cannot be disclosed if there are fewer than 9 cases.
